# Study Protocol: Phase I Dose Escalation Study of Oxaliplatin, Cisplatin and Doxorubicin Applied as PIPAC in Patients with Peritoneal Metastases

**DOI:** 10.3390/ijerph18115656

**Published:** 2021-05-25

**Authors:** Manuela Robella, Paola Berchialla, Alice Borsano, Armando Cinquegrana, Alba Ilari Civit, Michele De Simone, Marco Vaira

**Affiliations:** 1 Unit of Surgical Oncology, Candiolo Cancer Institute, Fondazione del Piemonte per l’Oncologia—IRCCS, 10060 Candiolo, Italy; alice.borsano@ircc.it (A.B.); armando.cinquegrana@ircc.it (A.C.); alba.ilari@ircc.it (A.I.C.); michele.desimone@ircc.it (M.D.S.); marco.vaira@ircc.it (M.V.); 2 Department of Clinical and Biological Sciences, University of Turin, 10124 Turin, Italy; paola.berchialla@unito.it

**Keywords:** cisplatin, doxorubicin, oxaliplatin, dose escalation, phase I, PIPAC, peritoneal carcinomatosis

## Abstract

Pressurized Intra-Peritoneal Aerosol Chemotherapy (PIPAC) is a novel laparoscopic intraperitoneal chemotherapy approach offered in selected patients affected by non-resectable peritoneal carcinomatosis. Drugs doses currently established for nebulization are very low: oxaliplatin (OXA) 120 mg/sm, cisplatin (CDDP) 10.5 mg/sm and doxorubicin (DXR) 2.1 mg/sm. A model-based approach for dose-escalation design in a single PIPAC procedure and subsequent dose escalation steps is planned. The starting dose of oxaliplatin is 100 mg/sm with a maximum estimated dose of 300 mg/sm; an escalation with overdose and under-dose control (for probability of toxicity less than 16% in case of under-dosing and probability of toxicity greater than 33% in case of overdosing) will be further applied. Cisplatin is used in association with doxorubicin: A two-dimensional dose-finding design is applied on the basis of the estimated dose limiting toxicity (DLT) at all combinations. The starting doses are 15 mg/sm for cisplatin and 3 mg/sm for doxorubicin. Safety is assessed according to Common Terminology Criteria for Adverse Events (CTCAE version 4.03). Secondary endpoints include radiological response according to Response Evaluation Criteria in Solid Tumor (version 1.1) and pharmacokinetic analyses. This phase I study can provide the scientific basis to maximize the optimal dose of cisplatin, doxorubicin and oxaliplatin applied as PIPAC.

## 1. Introduction

Peritoneal carcinomatosis (PC) is both a consequence of different primary tumors, synchronous or metachronous, and the clinical presentation of primitive peritoneal neoplasms. Despite significant recent advances in the management of peritoneal carcinomatosis, this diagnosis still is linked frequently to a poor prognosis. The unfavorable outcome is often accompanied by clinical symptoms that dramatically impact on quality of life and represent a real challenge for the managing health care provider.

Curative approach is, unluckily, reserved to a small minority of patients amenable to combined procedures based on cytoreductive surgery and locoregional treatments. The majority of patients is still nowadays treated by palliative approach.

### 1.1. The Failure of Systemic Treatment

The treatment of PC by palliative systemic chemotherapy (sCT) is still, nowadays, often the standard of care. Some cheering improvement in survival are recorded in PC from colonic cancer in which median survival for non-surgical amenable patients raised from six to 24 months by novel drugs agents, as FolfOX/FolfIRI ± bevacizumab [1]. In other PC, such as from gastric cancer, results are not so encouraging: literature reported median overall survival ranging from 4 to 13 months [2,3]. In ovarian cancer, intravenous chemotherapy with platinum compounds, taxanes, anthracyclines, gemcitabine, topotecan and trabectedin in various combinations and sequences are the mainstay of recurrence treatment. These regimens achieve median overall survival rates after the first, second, third, fourth and fifth relapse of 17.6 (95% CI 16.4–18.6), 11.3 (10.4–12.9), 8.9 (7.8–9.9), 6.2 (5.1–7.7) and 5.0 (3.8–10.4) months, respectively [4]. It is remarkable that the intraperitoneal availability of drugs by sCT is low; consequentially, the systemic treatment is often inefficient in bulky disease. Furthermore, the cumulative toxicity of intravenous repeated chemotherapeutic regimens is responsible for progressive decrease of patients’ compliance to therapy.

### 1.2. The Failure of the Intraperitoneal Chemotherapy

Intraperitoneal chemotherapy (IPC) has the potential to improve drug delivery to the tumor with generally accepted systemic side effects [5]. The rationale of this approach is represented by the possibility to consider the peritoneal cavity as a “pharmacologic sanctuary”, due to the presence of the peritoneal-plasma barrier that allows a high drug concentration in the abdominal cavity associated to minimal leakage towards systemic circulation. IPC is reported to be effective but is still burdened by pharmacological limitation, such as low homogeneity in drug distribution in the abdominal cavity [6] and technical problems like the high complication rate related to the intraperitoneal catheter (infections, obstruction, bleeding, dislocation): only 40% of patients are able to complete the expected chemotherapy cycles [7,8]. Furthermore, the poor drug penetration into peritoneal bulky disease (and adhesions-entrapped tumor nodules) in intraperitoneal administration is responsible for mediocre results if IPC is not preceded by optimal cytoreductive surgery [9].

### 1.3. PIPAC as a Promising Intraperitoneal Chemotherapy Delivery Technique

PIPAC takes advantage of the physical properties of gas and pressure avoiding the pharmacokinetic limitations of IPC [10]: under-pressure application and drug micronization enhance drugs uptake, peritoneal distribution and penetration depth [11,12,13].

Based on animal experimental data, PIPAC has been tested in patients with recurrent peritoneal carcinomatosis: It has been administered alone or after systemic fluorouracil [13,14,15]. Concomitant systemic treatment is possible with most used regimens, considering no systemic chemotherapy for two weeks before and one week after PIPAC procedure.

Two intraperitoneal regimens are used for PIPAC procedures: cisplatin in combination with doxorubicin and oxaliplatin as monotherapy. At least three PIPAC procedures are done at six- to eight-week intervals, but treatment can be pursued depending on disease response and patient tolerance.

So far, the dosages of these drugs have been set at approximately 20% of the dose used in HIPEC. Only one phase 1 study increased the doses of cisplatin and doxorubicin applied as PIPAC, setting them to a dosage still too low—10.5 mg/sm and 2.1 mg/sm, respectively [16]. Similarly, the oxaliplatin dose used for PIPAC has recently been the subject of a dose escalation study reporting that the recommended phase 2 dose should be 120 mg/sm [17].

The feasibility, safety and tolerance of repeated PIPAC treatment are confirmed by retrospective and prospective studies. Limited hepatic and renal toxicity are reported, associated to acceptable local toxicity: nausea and diffuse abdominal pain are the most complained complications. No acute or cumulative renal, gastrointestinal and hepatic toxicity are described [18,19,20]. Furthermore, surgical complications are rare. Whereas no mortality is observed in prospective trials, the mortality in retrospective studies is 2.7% [21].

At least, PIPAC has been shown to be safe regarding occupational health aspects such as operation theatre air contamination with aerosol chemotherapy particles [22].

Considering the efficacy of the procedure, not only retrospective studies, but also phase 2 trials described PIPAC as treatment able to induce regression of peritoneal nodules. Clinical response is reported in 62–88% of patients with ovarian cancer, in 50–91% of patients with gastric cancer, in 71–86% of colorectal cancer and 67–75% of peritoneal mesothelioma [21]. Moreover, in patients with advanced peritoneal carcinomatosis, PIPAC has been able to improve quality of life by up to 89% [23].

On the basis of the cited literature, this phase 1 study aims to determine the dose-related safety profile and tolerability of PIPAC with cisplatin, doxorubicin and oxaliplatin by assessment of dose-limiting toxicities and adverse events.

## 2. Materials and Methods

This is a prospective, single center, open-label, non-randomized, two-arm study.

The trial was originally designed in 2015 as a single arm study with repeated dose targeting patients not amenable to standard systemic chemotherapy. This approach significantly compromised the progress of the study since the majority of patients presented general clinical conditions suitable for a systemic treatment. The protocol was, therefore, modified, through an amendment, by creating two study arms:

- Cohort A: patients receiving standard systemic chemotherapy cycles in association with PIPAC.
- Cohort B: patients ineligible to receive standard systemic chemotherapy who will be treated using the PIPAC procedure alone. A dose escalation design was planned for this arm.

Ethics approval was obtained according to the guidelines of the Declaration of Helsinki and approved by the Ethics Committee of Candiolo Cancer Institute, FPO—IRCCS (EudraCT number 2015–000866-72 version 3.0—4 February 2018) and by the Italian drug agency (AIFA—Agenzia Italiana del FArmaco—5 April 2018); the trial is registered on ClinicalTrials.gov, number NCT02604784.

### 2.1. PIPAC Administration

**Figure 1:**
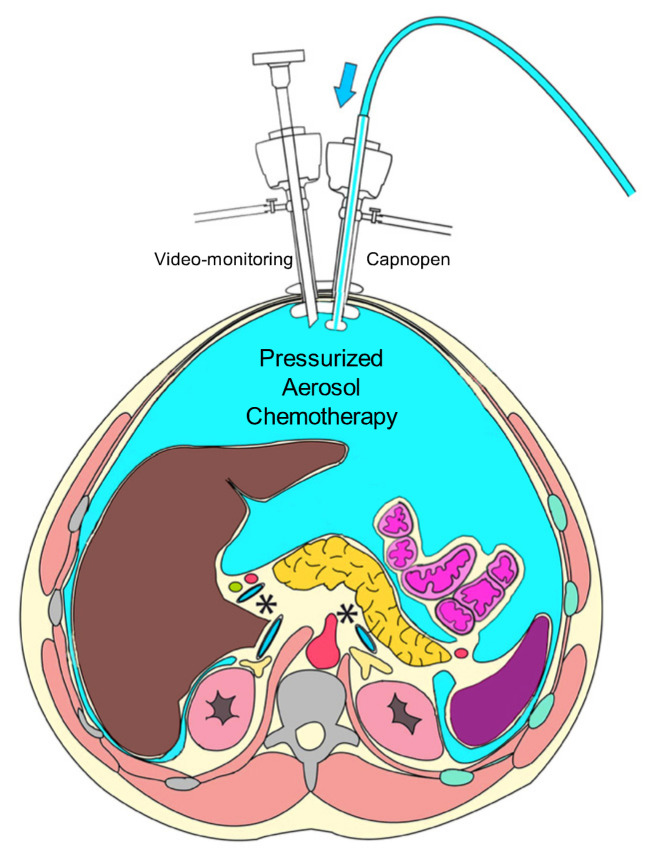
Schematic of single-port PIPAC set-up. The nebulizer connected to a standard injector and a laparoscope are inserted through a Quadport+ platform.

PIPAC procedure is performed as previously described [24]. Briefly, an open access with a midline 5–6 cm incision is performed and a single-port platform (QuadPort+ Olympus) is positioned according to our original technique (Figure 1). A 12 mmHg CO2 pneumoperitoneum is inflated. Ascites is removed if present and the amount documented. Video documentation is started; PC extent is evaluated according to the Peritoneal Cancer Index (PCI) and multiple peritoneal biopsies are taken for histological examination and baseline tissue drug concentration detection. A nebulizer (Capnopen^®^, Capnomed, Villingendorf, Germany) is connected to a high-pressure injector and inserted into the peritoneal cavity; the tightness of the abdomen is documented with a CO_2_ zero-flow. The camera and the nebulizer are maintained in position by a self-retaining retractor (Thompson). The pressurized aerosol containing cisplatin and doxorubicin or oxaliplatin at the respective dose according to the dose escalating design is applied through the nebulizer. The flow rate is set at 30 mL/min and the maximal upstream pressure is 200 PSI. The injection is remote-controlled in order to avoid occupational exposure. The capnoperitoneum is then maintained for 30 min at 37 °C. At the end, the aerosol is exsufflated through two sequential micro-particle filters into the air-waste system of the hospital. Single-port platform is removed; no abdominal drain tube is applied. Nasogastric tube and urinary catheter are removed at the end of the operation.

### 2.2. Study Population

Eligible patients should present peritoneal mesothelioma, primary peritoneal tumor or unresectable peritoneal metastasis from ovarian, gastric, intestinal and appendiceal cancer. Suitability and eligibility of the patient have to be validated by a multidisciplinary team.

### 2.3. Inclusion Criteria

Patients eligible for recruitment must meet all of the following criteria:

- Unresectable peritoneal metastasis on peritoneal cytology/histology;
- Age between 18 and 80 years;
- Eastern Cooperative Oncology Group (ECOG) performance status ≤2;
- Adequate liver function [AST/SGOT and/or ALT/SGPT ≤2.5 × ULN (upper limit of the normal range) or ≤5 × ULN if liver metastases are present, serum bilirubin ≤1.5 × ULN];
- Adequate renal function (serum creatinine ≤ 1.5 × ULN or creatinine clearance >50 mL/min);
- Cardiac and pulmonary function preserved;
- Adequate bone marrow function [absolute neutrophil count (ANC) ≥ 1.5 × 109/L, hemoglobin (Hb) ≥9 g/dL, platelets (PLT) ≥100 × 109/L];
- Total recovery or a CTCAE grade ≤1 from all adverse clinical events of previous chemotherapy, including surgery and radiotherapy, except for alopecia;
- No chemotherapy/major surgery in the last four weeks prior to the PIPAC procedure;
- Written informed consent signed.

### 2.4. Exclusion Criteria

Any of the following is considered an exclusion criterion:

- Extra abdominal metastatic disease (with the exception of isolated pleural carcinomatosis);
- Bowel obstruction;
- History of allergic reactions to cisplatin/doxorubicin/oxaliplatin or their derivatives;
- Severe renal failure, myelosuppression, severe hepatic failure, severe heart failure, recent myocardial infarction, severe arrhythmia;
- Immunosuppressed patients, undergoing immunosuppressive therapy;
- Previous treatment with reaching the maximum cumulative dose of doxorubicin, daunorubicin, epirubicin, idarubicin and/or other anthracyclines and anthracenedions;
- Pregnancy;
- Patients of both sexes with reproductive potential who refuse to use an adequate method of contraception;
- Major surgery or systemic chemotherapy less than four weeks prior to PIPAC procedure.

### 2.5. Study Objective

The primary endpoint of the study is to determine the incidence of dose-limiting toxicity of PIPAC with cisplatin, doxorubicin or oxaliplatin (according to the primary pathology) performed once in patients with peritoneal carcinomatosis. Toxicity will be graded using the National Cancer Institute (NCI) Common Terminology Criteria for Adverse Events (CTCAE) version 4.03.

Secondary endpoints will be: pharmacokinetics of cisplatin, doxorubicin and oxaliplatin as pressurized aerosol by the intraperitoneal route; evaluation of the clinical tumor response based on RECIST criteria (version 1.1) after PIPAC.

### 2.6. Statistical Design

Cisplatin and doxorubicin will be used in patients with peritoneal carcinomatosis of ovarian and gastric origin and in primary tumors of the peritoneum. A dose escalation model based on a two-agents combination design published by Riviere is adopted [25]. It is an extension of the Continual Reassessment Method in case of two-dimensional dose-escalation, which identifies the MTD of the combination of cisplatin and doxorubicin based on the probability of dose limit toxicity (DLT) of each combination of the two agents.

An empirical logistic model in a Bayesian framework is adopted. The model of the probability of toxicity at a given dose combination is defined as following:

*logit*(*π*(*d*_1*j*_,*d*_2*k*_*α*,*β*_1_,*β*_2_,*β*_3_) = *α* + *β*_1_*d*_1,*j*_ + *β*_2_*d*_2,*k*_ + *β*_3_*d*_1,*j*_*d*_2,*k*_ (1)

where *β*_1_ represents the toxicity effect of agent 1 (cisplatin), *β*_2_ of agent 2 (doxorubicin), and *β*_3_ is the interaction effect potentially due to the combination of the agents.

For parameters α and *β*_3_, a vague normal prior distribution centered at 0 to indicate that a priori either positive or negative values are favored, letting the observed number of patients with toxicity driving the posteriori distribution. For parameters (*β*_1_, *β*_2_), an exponential distribution with mean 1 is chosen, based on the consideration that (*β*_1_, *β*_2_) should numerically be in a neighborhood of 1.

The working model is defined by dose levels *d*_1*,j*_ for cisplatin and *d_2,k_* for doxorubicin, which have been identified on the basis of a modified Fibonacci series.

The first cohort of participants will be treated with cisplatin 15 mg/sm body surface in 150 mL NaCl 0.9% and doxorubicin 3 mg/sm (cohort 1); the following cohort will receive CDDP 30 mg/sm and DXR 6 mg/sm (cohort 2) and the third cohort CDDP 50 mg/sm and DXR 10 mg/sm (cohort 3). Dose escalation will be continued by the protocol according to the probability model up to a maximum of CDDP 100 mg/sm (that is the dose currently used in HIPEC procedure) and DXR 30 mg/sm.

The different dose increases will be adopted as reported in Table 1.

The recommended doses of both agents are those of the dose level combination associated with a probability of DLT closes to the DLT probability target at 25%. An escalation with overdose and under-dose control (for probability of toxicity less than 16% in case of under-dosing; and probability of toxicity greater than 33% in case of overdosing) is further applied. No skipping dose is allowed, nor intra-patient dose escalation.

As a stopping rule, the maximum number of 42 patients is considered.

Oxaliplatin will be used in patients presenting peritoneal carcinomatosis of intestinal origin.

An extension of the Continual Reassessment Method based on a two-parameter probability model proposed by Neuenschwander will be used to identify the recommended dose of oxaliplatin [26].

The first cohort of patients will be treated with oxaliplatin 100 mg/sm body surface in 150 dextrose solution 5% (cohort 1); the following cohort will receive oxaliplatin 135 mg/sm (cohort 2) and 155 mg/sm for the third one (cohort 3).

After each cohort is assessed, a probability of toxicity, given the data observed (i.e., the number of patients who experienced toxicity), is computed on the basis of the following 2-parameters logistic regression model

*logit*(*π*(d*_j_;*α*,*β*) = *logα* + *β* × d*_j_, *α*,*β* > 0 (2)

where the parameters (*α*, *β*), which are positive valued, ensure a monotonically increasing dose-toxicity relationship; d*_j_ is a dose standardized to a reference dose, so that log *α* can be interpreted as the log-odds of toxicity when d*_i_ is the reference dose.

Parameters (*α*, *β*) will be calibrated to reflect information about toxicities, corresponding to assuming a toxicity probability of oxaliplatin at the maximum dose of 460 mg equal to 25% and a toxicity probability at the starting dose of 120 mg equal to 10. Calibration of the parameters will be carried out according to the approach proposed in Thall [27], assuming a zero a priori correlation.

An escalation with overdose and under-dose control (for probability of toxicity less than 16% in case of under-dosing; and probability of toxicity greater than 33% in case of overdosing) is further applied. No skipping dose is allowed, nor intra-patient dose escalation.

The dose escalation design of oxaliplatin is presented in Table 2.

### 2.7. Outcome Measures

Patient demographics, clinical features, surgical treatment details, AEs, clinical laboratory evaluations and safety data of cisplatin, doxorubicin and oxaliplatin administered as PIPAC will be collected.

Toxicity will be graded using CTCAE version 4.03. DLT is defined as any severe chemotherapy-related grade ≥3 toxicity. Patients will be assessed on day 0, 1, 2 (until the date of discharge), 15, 28 for toxicities, adverse events, hematology and chemistries.

Clinical response will be assessed with contrast enhanced computed tomography according Response Evaluation Criteria in Solid Tumor (RECIST v. 1.1).

Doxorubicin, cisplatin and oxaliplatin plasma levels will be assayed with blood samples drawn prior to and 30, 60, 120 min and 6, 12, 24 h after PIPAC procedure at each dose level.

## 3. Discussion

Peritoneal carcinomatosis is still nowadays one of the toughest oncological challenge, with a poor prognosis due to a weak response to systemic treatments.

The intraperitoneal chemotherapy administration was found to be effective [5], but still burdened by complications related to the infusion catheter which have always limited its repeatability. Moreover, its effectiveness is restricted by drug intraperitoneal distribution and tumor penetration [28].

PIPAC seems to have achieved a better distribution and penetration taking advantage of the pressurized drug micronization and aerosolization, while maintaining the virtues of the standard intraperitoneal administration (lower systemic toxicity and higher tissue penetration as compared to systemic chemotherapy).

Not only retrospective studies, but also phase II trials demonstrated promising results in terms of efficacy. A German phase II study reported a histological tumor regression and PC Index improvement in 26/34 (76%) and in 26/34 (76%) patients with advanced ovarian cancer submitted to three PIPAC procedures [18]. Another Italian study with sixty-three patients with peritoneal carcinomatosis of different origins reported an objective response in 14 patients (35%). In this study, PIPAC was often associated to systemic chemotherapy: the combined treatment did not induce significant hepatic and renal toxicity and the author suggested it as valid therapeutic option in patients with advanced peritoneal disease [19]. A further phase 2 study in patients with peritoneal disease from gastric cancer reported a radiological complete, partial response or stable disease in 40% of patients [29].

To date, the promising early results demonstrated encouraging response rates to PIPAC approach, which came along with expected benefit survival and a reduction of symptoms related to the disease diffusion. Higher drugs doses could improve efficacy and make PIPAC a promising treatment for advanced peritoneal diseases or refractory ascites.

This is one of the very few phase 1 studies about PIPAC born from the hypothesis that higher drug doses could be safely administered as pressurized intraperitoneal aerosol [30,31].

High dose of platinum-based chemotherapy given by intraperitoneal route are reported to be well tolerated [32]; moreover, the doses of cisplatin, oxaliplatin and doxorubicin used in HIPEC procedure are usually higher [33,34,35].

Local drug administration limits systemic toxicities thanks to the possibility to consider the peritoneum as a sanctuary in which the peritoneal layer acts as barrier; with cisplatin 100 mg/sm administered via HIPEC, the maximum drug concentration detected in plasma is 1.71 µg/mL [35].

Platinum based intraperitoneal chemotherapy have dose-dependent efficacy [36]: higher intraperitoneal doses result in higher intratumoral concentrations with a consequent higher efficacy. Moreover, the combination of CDDP and DXR appears to be one of the most effective available regimens with acceptable local-regional toxicity [37]. A dose escalation study is, therefore, essential to evaluate cisplatin, doxorubicin and oxaliplatin pharmacokinetics and relative tissue concentrations.

## 4. Conclusions

This phase I study aims to identify the recommended doses of oxaliplatin, cisplatin and doxorubicin applied as PIPAC through an evidence-based approach. The results of this study could be the starting point of subsequent phase 2 studies aimed to evaluate and maximize the effectiveness of this promising technique.

## Figures and Tables

**Table 1:** Cisplatin and doxorubicin dose escalation design.

Level	Cisplatin (CDDP)	Doxorubicin (DXR)
1	15 mg/sm	3 mg/sm
2	30 mg/sm	6 mg/sm
3	50 mg/sm	10 mg/sm
4	67 mg/sm	13 mg/sm
5	88 mg/sm	18 mg/sm
6	93 mg/sm	23 mg/sm
7	100 mg/sm	30 mg/sm

**Table 2:** Oxaliplatin dose escalation design.

Level	Oxaliplatin (OXA)
1	100 mg/sm
2	135 mg/sm
3	155 mg/sm
4	180 mg/sm
5	200 mg/sm
6	235 mg/sm
7	270 mg/sm
8	300 mg/sm

## Data Availability

Not applicable.
